# Histopathological findings of biodegradable polymer sirolimus eluting stent 7 years after stent implantation

**DOI:** 10.1016/j.jccase.2025.01.007

**Published:** 2025-02-18

**Authors:** Yo Kawahara, Sho Torii, Yasutomo Sekido, Gaku Nakazawa

**Affiliations:** a Department of Cardiology, Isehara Kyodo Hospital, Isehara, Kanagawa, Japan; b Department of Cardiology, Tokai University School of Medicine, Isehara, Kanagawa, Japan; c Department of Pathology, Isehara Kyodo Hospital, Isehara, Kanagawa, Japan; d Department of Cardiology, Kindai University School of Medicine, Sayama, Osaka, Japan

**Keywords:** Neoatherosclerosis, Drug-eluting stent, Very late stent thrombosis

## Abstract

This report analyzes a biodegradable polymer coated drug-eluting stent (DES), Ultimaster (Terumo, Tokyo, Japan), seven years after implantation in a 73-year-old man who died from acute myocardial infarction after discontinuing his medications. Autopsy revealed no in-stent thrombosis or restenosis. Two stents exhibited neoatherosclerosis with calcifying necrotic core and foamy macrophages, indicating a lesser risk of very late stent thrombosis. The findings support the notion that third-generation DES might result in healthier long-term vessel healing and reduced neoatherosclerosis compared to earlier generations, consistent with prior animal studies. This suggests a sustained benefit and safety of the biodegradable polymer coated DES over an extended period.

**Learning objectives:**

A 65-year-old male received three 3rd generation biodegradable polymer coated drug-eluting stents (BP-DES), Ultimaster (Terumo, Tokyo, Japan), during percutaneous coronary intervention. Seven years post-implantation, post-mortem histopathological analysis revealed well-healed arterial tissue with near-complete endothelialization and minimal neoatherosclerosis. No significant inflammation or late stent thrombosis was observed, with stent struts embedded in the neointima, indicating favorable long-term vessel healing. This case underscores the long-term biocompatibility of BP-DES, highlighting reduced risks of late stent thrombosis and neointimal hyperplasia over extended follow-up periods.

## Introduction

Stent thrombosis (ST) is a significant complication in patients who receive drug-eluting stents (DES). Previous registries utilizing optical coherence tomography (OCT) have shown that the primary cause of ST within the first year after stent implantation is predominantly uncovered struts [[1], [2], [3], [4]]. In contrast, neoatherosclerosis has been identified as the main cause of very late ST [1,5,6]. Abluminally coated biodegradable polymer DES (BP-DES), often referred to as third-generation DES, was developed with the aim of reducing the risk of neoatherosclerosis. Despite the promise of third-generation DES, there is limited data on their long-term pathological effects, underscoring the importance of this investigation.

## Case report

This case report presents a rare histopathological examination of a third-generation biodegradable polymer coated sirolimus-eluting stent (BP-SES), Ultimaster (Terumo, Tokyo, Japan), 7 years post-implantation in a 73-year-old male patient with a complex medical history, including diabetes mellitus, hypertension, peripheral arterial disease, and dementia. Autopsy findings revealed insights into the long-term effects of the BP-SES, an important addition to the limited understanding of neoatherosclerosis in BP-SES beyond 5 years.

The patient had received three BP-SESs in three major epicardial coronary arteries seven years earlier for chronic coronary syndrome. He had self-discontinued his medications approximately two years before presenting with acute chest pain. Blood tests demonstrated poor control of risk factors, with hemoglobin A1c at 11.6 % and low-density lipoprotein/non-high-density lipoprotein cholesterol levels of 108/123 mg/dL. Although diagnosed with acute myocardial infarction with ST elevation on leads V2–6 and elevated cardiac enzymes (creatine phosphokinase: 889 IU/L, troponin T: 3.2 ng/ml), the patient declined invasive treatment and subsequently died the next day.

**Fig. 1:**
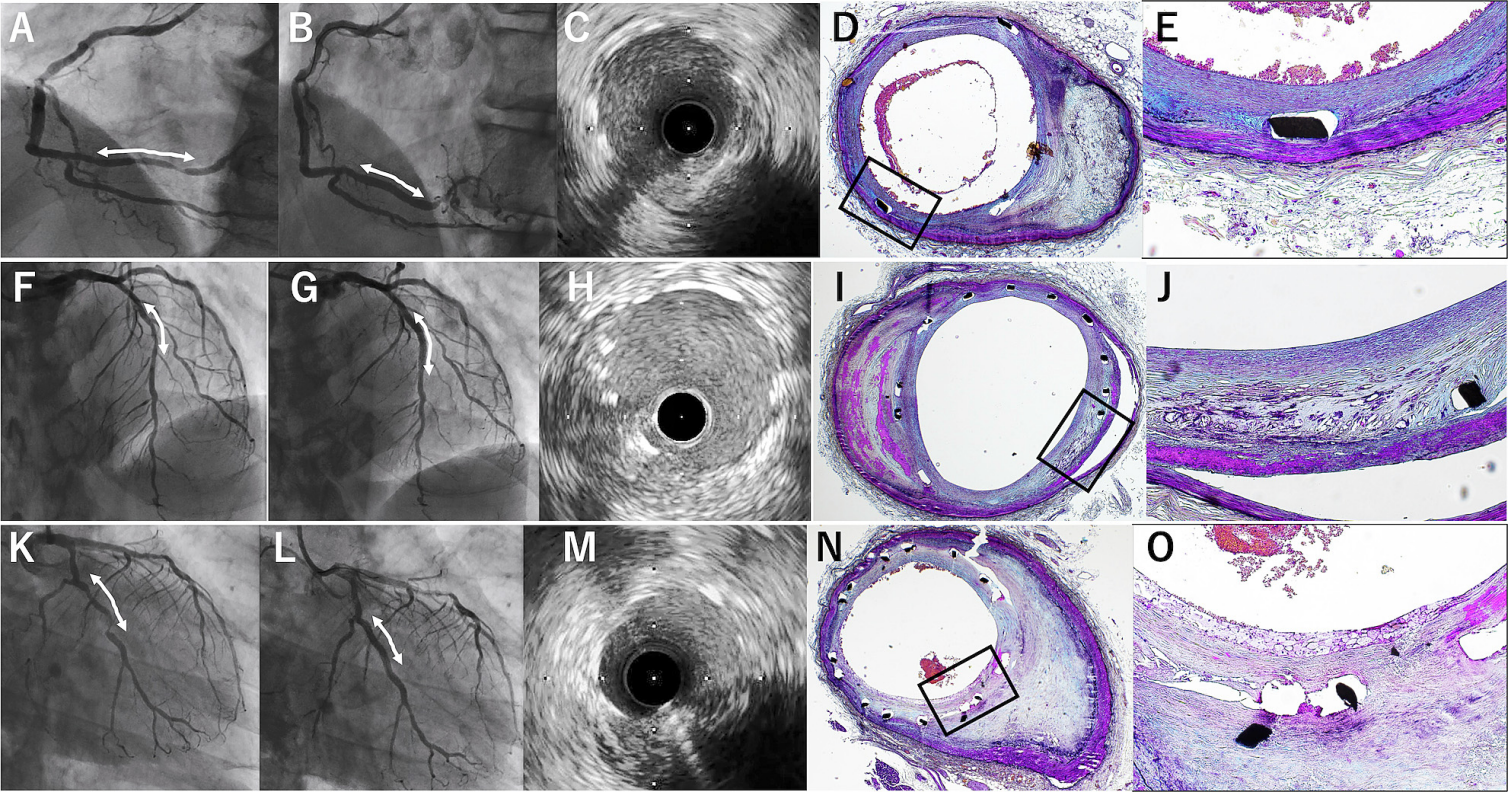
Co-registered images of angiography, intravascular ultrasound (IVUS) at the initial procedure and histological sections 7 years after biodegradable polymer coated sirolimus eluting stent implantation. Pre- and post-angiography images, IVUS findings after stent implantation, and histological sections at low and high magnification, stained with Movat Pentachrome, are shown for the right coronary artery (RCA, 2.75 mm/24 mm, panels A–E), left anterior descending artery (LAD, 2.75 mm/24 mm, panels F–J), and left circumflex artery (LCX, 2.5 mm/24 mm, panels K–O). Histopathological examination revealed healthy neointima on the moderately eccentric calcified plaque in the RCA (D and E), neoatherosclerosis with calcifying necrotic cores on the fibrous plaque in the LAD (I and J), and neoatherosclerosis with superficial foamy macrophage infiltration over an eccentric calcified plaque in the LCX (N and O).

Pathological examination of the myocardium demonstrated acute myocardial infarction in the antero-septal area, classified as cardiac death. Stented lesion showed no signs of in-stent thrombosis or restenosis (Fig. 1). The right coronary artery was fully endothelialized, indicating optimal healing over the underlying moderate, eccentrically calcified plaque. The left anterior descending artery showed neoatherosclerosis with small, localized calcifying necrotic cores on an underlying fibrous plaque, while the left circumflex artery presented with neoatherosclerosis and superficial foamy macrophage infiltration, but no necrotic core, on an eccentric calcified plaque. Both lesions showed neoatherosclerosis; however, they were relatively benign compared to neoatherosclerosis with necrotic cores, indicating a lower risk of very late stent thrombosis (VLST).

The presence of neoatherosclerosis in two of the three BP-SESs, with features such as calcifying necrotic cores and surface foamy macrophage infiltration, indicated a relatively lower VLST risk. Interestingly, the underlying plaques in lesions with neoatherosclerosis exhibited moderate calcification and stable fibrous plaques. Conversely, recent pathological evaluations have suggested that the neointimal coverage rate differs between complex lesions (culprit lesions of acute coronary syndrome or surface calcification) and simpler lesions [7]. Further pathological studies are needed to investigate the influence of underlying plaque characteristics on arterial healing after DES implantation.

Prior animal studies posited that the third-generation DES with abluminally coated biodegradable polymer, might be associated with healthier neointima formation, resulting in reduced risk of neoatherosclerosis compared to second-generation DES [[8], [9], [10], [11], [12], [13]]. This case supports the hypothesis that third-generation DES can maintain healthier healing profiles even after 7 years of implantation, aligning with animal studies that might suggest a lesser risk of neoatherosclerosis with the third-generation DES.

## Statement of consent

The authors confirm that written consent for submission and publication of this case report including images and associated text has been obtained from the patient in line with COPE guidance.

## Declaration of competing interest

No financial support was provided for this manuscript. All authors have no relationships relevant to the contents of this paper to disclose.
